# Global Data and Digital Public Health Leadership for Current and Future Pandemic Responses

**DOI:** 10.3389/fdgth.2021.632568

**Published:** 2021-01-28

**Authors:** Bandar Al Knawy

**Affiliations:** Health Affairs, Ministry of National Guard and King Saud Bin Abdulaziz University for Health Sciences, Riyadh, Saudi Arabia

**Keywords:** COVID-19, digital public health, leadership, pandemic (COVID-19), public health

COVID-19 has created, and continues to create, a deep and permanent scar, with individuals, cities, and countries still reeling from its impact. Whilst the most visible damage is the loss of many thousands of lives, the personal, social, and economic impacts—such as on mental health and job security—are only just becoming apparent (1).

Digital health (DH) refers to the use of information technology and/or electronic communication tools, services, and processes to deliver healthcare services or improve health. DH has been a critical component of the international public health (PH) response to COVID-19 and has maintained health and care delivery through telehealth solutions (2), using technology to improve public compliance with infection control measures, using data and artificial intelligence in surveillance and contact tracing systems (3, 4), and digital modeling (5). Recognizing this central role for DH in the pandemic, the Riyadh Global Digital Health Summit was conceptualized as a means to facilitate global dialogue between PH experts, DH leaders, researchers, and health and care system leaders. The 2020 Saudi Presidency for the G20 provided a perfect opportunity to launch this Summit as an associated event and included international experts from Asia, Africa, Australasia, North America, and Europe representing international organizations such as the European Center for Disease Prevention and Control and the WHO. The summit aimed to promote DH technologies and their role in enabling PH solutions in the fight against pandemics across many domains including monitoring, surveillance, detection, prevention, and mitigating the disease's impact on health and care system delivery. It also aimed to foster responsible and well-governed use of data to fight pandemics and, just as importantly, to maintain public trust and best cybersecurity practices. The summit concluded with the “Riyadh Digital Health Declaration,” which established nine key recommendations, core principles, and priorities for leveraging digital technologies to combat the current and future impact of the COVID-19 pandemic (6).

The Summit attracted over 135,000 virtual registrants and 300,000 non-registered viewers across a range of disciplines: 45,000 and 35,000 nurses and doctors, 15,000 and 11,000 pharmacists and allied health professionals, and 2,500 and 1,400 health informatician and PH experts. The Summit was one of the largest virtual health conferences of the year, and certainly the largest to focus on interactions between DH and PH.

Attendees interacted with the organizers and session chairs and co-chairs through an electronic platform. Live virtual dialogue between the speakers, chairs, and co-chairs was encouraged through interactive questionnaires, reactions, and online survey questions integrated into the discussions after presentations. These data were analyzed to feed into the final Declaration. After filtering out greetings and technical support questions, 2,641 questions and comments underwent natural language processing (NLP) to extract the main themes and correlated terms from unstructured text using R v3.5 software with the *tm v0.7-7* package. Key topics were modeled using unsupervised learning (latent Dirichlet allocation).

The most important issue participants said they would like addressed as a Summit outcome was addressing technology-related challenges by advancing data sharing and encouraging and facilitating low- and middle-income countries to adopt advanced technologies. The second most pressing issue was monitoring, surveillance, detection, and prevention, particularly at large events. The main messages, insights, and reflections taken from the event by participants were the role of technologies in DH (analysis, visualization, decision-making, time, alerts, and prediction); privacy, ethics, and cybersecurity; the future of telehealth in the post-pandemic era; and the need for collaboration between institutions, academics, businesses, and leaders.

The high attendance at the Summit reflected the importance and timeliness of the subject and also how technology can facilitate a near-reality experience of international conferences, accommodating huge numbers of participants beyond that usually possible for traditional in-person attended meetings. By bringing together the expert faculty and diverse participants, the following lessons were learned:

- DH has been essential to national responses to the COVID-19 pandemic and has boosted the use and acceptance of DH solutions by patients, clinicians, and policy makers (3).
- Digital technologies have significantly changed the landscape of “nudging” the public through novel crisis communication (7), but accountable and evidence-based digital nudging technologies are essential for full public acceptance and adoption.
- Digital epidemiology tools are beneficial for tracking and managing epidemics but must be considered within a wider and comprehensive PH infrastructure (8). This is a rapidly progressing field that will compliment traditional epidemiology to understand patterns of human disease (8). The reliance on existing data sources established for non-epidemiological purposes creates unavoidable challenges and biases in the data. These tools will be most beneficial in communities/countries when based on strong ethical and legal principles.
- The same way the world mastered weather forecasting using predictive modeling, we need to master disease/pandemic surveillance through enhanced predictive modeling (9). Digital epidemiology and infodemiology must be an integral component of PH responses to deliver positive health outcomes and mitigate all associated risks. The technological toolkit for the predictive modeling of pandemics is evolving quickly, and newer directions include improved short- and long-term predictions, inclusion of diverse data sources, as well as considering dynamic factors such as mobility, mask use, air quality, and seasonality.
- The new post-COVID-19 “normal” requires new ways of working together and new multisector partnerships and collaboration to ensure that the global response minimizes the negative impact and mortality risks of future pandemics (10). Health policy needs to ensure national and international capacity to respond rapidly and appropriately to pandemics. Harnessing the potential of DH systems is essential to ensuring that policy decisions during a pandemic are informed by real-time information on the severity and trajectory of the pandemic, especially for key at-risk groups such as the elderly or those with comorbidities.
- Citizens should be recognized as decision makers. However, they also need confidence that their privacy and data rights will be respected and knowledge of how their data will be used in the fight against COVID-19, whether in deciding to download a tracing app, consenting to provide data for research, or having confidence that open data is indeed anonymous (8).

In this age of continuous connection and digitization, pandemic responses require a strong and unified global leadership. The lessons learned should not be wasted. Every cloud has a silver lining, and COVID-19 has opened up new opportunities to connect, learn, innovate, and consider how to better fight communicable diseases. It has brought the importance of PH to the forefront as a critical global health security issue. DH has played a crucial role in the fight against COVID-19, and now is the time to leverage DH to deliver a global and unified approach to current and future disease outbreaks through a paradigm shift in *global digital public health leadership*.

The summit redefined DH as the very best of combining modern technologies, medicine, science, and innovation with data and PH to benefit the health and well-being of humanity. We should seize this momentum to modernize, innovate, redesign, and strengthen global health connectivity for the long haul. The Riyadh Digital Health Declaration provides a roadmap to guide this process toward DH, and there can be little doubt that health and care leadership around the globe are eager and calling for every nation's strong will to achieve this. It is now time to leverage this momentum and create a global movement to drive this new definition forward and sustain the global dialogue and stewardship. Innovative, transformative and sustainable digitization of PH is a global obligation.

It is encouraging that with the Summit's clear focus on DH in pandemics, the largest attendance groups were frontline healthcare workers. This is a significant and positive change and reflects the greater role played by technology in health and care delivery, as seen during this pandemic.

Governments should consider digital PH preparedness and response as a national security issue. Simultaneously, global biomedical science and technology research must become an integral component of the global health security alliance. The aftermath should accelerate not only the adoption but enforcement of the bigger scope of the global DH connectivity agenda, possibly at the level of the United Nations.

## Author Contributions

The author confirms being the sole contributor of this work and has approved it for publication.

## Conflict of Interest

The author declares that the research was conducted in the absence of any commercial or financial relationships that could be construed as a potential conflict of interest.
